# The Promise of Comprehensive Cancer Control

**Published:** 2009-09-15

**Authors:** Carol Friedman

**Affiliations:** Immunization Services Division, National Center for Immunization and Respiratory Diseases. Dr Friedman was with CDC’s Division of Cancer Prevention and Control during the writing of this article

In 1986, fifteen years after President Richard Nixon declared war on cancer, the National Cancer Institute (NCI) set an ambitious goal of reducing the cancer death rate by 50% by the year 2000 (1). However, by the mid-1990s, despite considerable investments in cancer prevention and control efforts by the Centers for Disease Control and Prevention (CDC), NCI, and the American Cancer Society, it became apparent that the goal would not be achieved. Cancer control leaders recognized the need for a more comprehensive approach to reducing the nation's cancer burden — one that involved partners and collaborative efforts among the many sectors affected by cancer.

In 1998, CDC funded 5 states and 1 tribal health board that had existing comprehensive cancer control plans to assess the feasibility of implementing their plans. The pilot project funded Colorado, Massachusetts, Michigan, North Carolina, Texas, and the Northwest Portland Area Indian Health Board and signaled the beginning of CDC's National Comprehensive Cancer Control Program (NCCCP). Since 1998, the number of programs participating in the NCCCP has grown from 6 to 65, including all 50 states, the District of Columbia, 7 tribes and tribal organizations, and 7 US-associated Pacific Islands/territories.

In this issue, we present 4 articles on the nationwide comprehensive cancer control movement, defined as an integrated and coordinated approach to reducing cancer incidence, morbidity, and mortality through prevention, early detection, treatment, rehabilitation, and palliation. Miller and colleagues describe early efforts by state and tribal coalitions to establish cancer control programs (2); Major and Stewart follow up with the story of the national program's first decade (3). Robinson and Williams describe how the Louisiana Comprehensive Cancer Control Program used a multilevel organizational approach to maintain cancer control activities in the aftermath of Hurricane Katrina (4). Jenkins et al detail the use of geocoding to analyze disparities in hospice care (5).

In 2008, the first decline in overall age-adjusted cancer incidence rates was reported (6). Despite lower cancer incidence and death rates, the actual number of Americans diagnosed with cancer continues to increase each year and is expected to reach 2.6 million by 2050 (7). According to the most current data, cancer has overtaken heart disease as the leading cause of death in 8 states: Alaska, Colorado, Maine, Minnesota, Montana, New Hampshire, Oregon, and Washington (8).

Improved early detection tools and medical treatments have increased the 5-year survival rates for patients diagnosed with many of the common cancers of childhood and adulthood. These patients will require additional medical services to improve their chances of survival and ensure their quality of life after a diagnosis. A comprehensive approach to cancer prevention and control that follows the cancer continuum from prevention to survivorship is needed. We hope that these articles will serve as a catalyst for cancer researchers, public health practitioners, and policy makers to continue to support and implement this collaborative nationwide movement to fulfill the promise of cancer prevention and control in all US communities.
